# Sex-based differences in myocardial perfusion imaging among diabetic Egyptian patients:a retrospective registry study

**DOI:** 10.21542/gcsp.2026.35

**Published:** 2026-08-31

**Authors:** Ahmed Ibrahim El-Desoky Khalil, Osama Mohamed Mohamed Alzebak, Amr Adel Alsayed, Hazem Mohamed Mansour

**Affiliations:** Department of Cardiology, Faculty of Medicine, Ain Shams University, Cairo, Egypt

## Abstract

**Background:** Diabetes mellitus (DM) markedly increases the risk of coronary artery disease (CAD), and myocardial perfusion imaging (MPI) is a key noninvasive tool for risk stratification. Sex-based differences in presentation, referral patterns, and diagnostic accuracy of MPI remain understudied, particularly in Middle Eastern populations with a high prevalence of diabetes. This study evaluated sex-based differences in the indications, results, appropriateness, and diagnostic accuracy of MPI among diabetic Egyptian patients.

**Methods:** This retrospective observational registry study included 1,000 diabetic adults (648 men, 352 women) who underwent gated SPECT-MPI at our university hospitals. Demographic data, risk factors, ECG findings, stress modalities, MPI results, coronary angiography data, and appropriate use criteria (AUC) were analyzed.

**Results:** Men were older than women (59.6 ± 8.0 vs. 57.0 ± 9.4 years, *p* < 0.001). Hypertension was more prevalent in women (84.1% vs. 67.6%, *p* < 0.001), whereas smoking was more common in men (30.4% vs. 0.3%, *p* < 0.001). Positive MPI results were more frequent in men (77.0% vs. 43.2%, *p* < 0.001), as were appropriate indications (79.3% vs. 53.4%, *p* < 0.001); inappropriate and uncertain indications were correspondingly more common in women. Reversible perfusion defects did not differ significantly by sex (23.6% in women vs. 21.2% in men, *p* = 0.523), whereas fixed defects were more prevalent in men (17.0% vs. 7.8%, *p* < 0.001). MPI accuracy relative to coronary angiography did not differ significantly between the sexes (91.7% in men vs. 86.3% in women, *p* = 0.103).

**Conclusion:** Substantial sex-based disparities exist in MPI referral patterns, test positivity, and appropriateness among diabetic Egyptians. Women are more likely to receive inappropriate referrals and to have a lower positive diagnostic yield, underscoring the need for sex-sensitive clinical protocols.

## Introduction

Ischemic heart disease (IHD) remains the leading cause of mortality worldwide, accounting for approximately 9 million deaths annually^1^. Among the many risk factors for IHD, diabetes mellitus (DM) is one of the most potent, conferring a two- to four-fold increased risk of cardiovascular events and death^2^. Patients with DM are not only more likely to develop myocardial ischemia but also experience higher rates of silent ischemia and adverse outcomes following acute coronary syndromes^3^.

The global burden of DM is considerable: an estimated 537 million adults were living with diabetes in 2021, a number projected to rise to 783 million by 2045^4^. Egypt ranks among the top ten countries worldwide for diabetes prevalence, with approximately 15.2% of the adult population affected^5^. This high prevalence, together with distinctive cultural and socioeconomic factors, makes the Egyptian population particularly important for cardiovascular research.

Myocardial perfusion imaging (MPI) using single-photon emission computed tomography (SPECT) is a valuable noninvasive tool for evaluating patients with known or suspected CAD^6^. In diabetic patients, MPI provides critical information about the presence and severity of ischemia, myocardial viability, and overall cardiac risk, guiding clinical decisions regarding medical therapy, revascularization, and further diagnostic testing^7^.

Sex-based differences in cardiovascular disease have gained increasing recognition over the past two decades^8^. Women with CAD often present with atypical symptoms, have a higher prevalence of nonobstructive CAD and coronary microvascular dysfunction, and are less likely than men to be referred for invasive procedures^9^. In the context of noninvasive testing, women are more likely to receive inappropriate referrals for MPI and to have lower rates of abnormal findings^10^. However, most of these studies have been conducted in Western populations, with limited data from Middle Eastern countries.

This study evaluated sex-based differences in MPI indications, results, appropriateness, and diagnostic accuracy among diabetic Egyptian patients undergoing MPI at a tertiary care academic center.

## Methods

### Study design and setting

**Figure 1.**
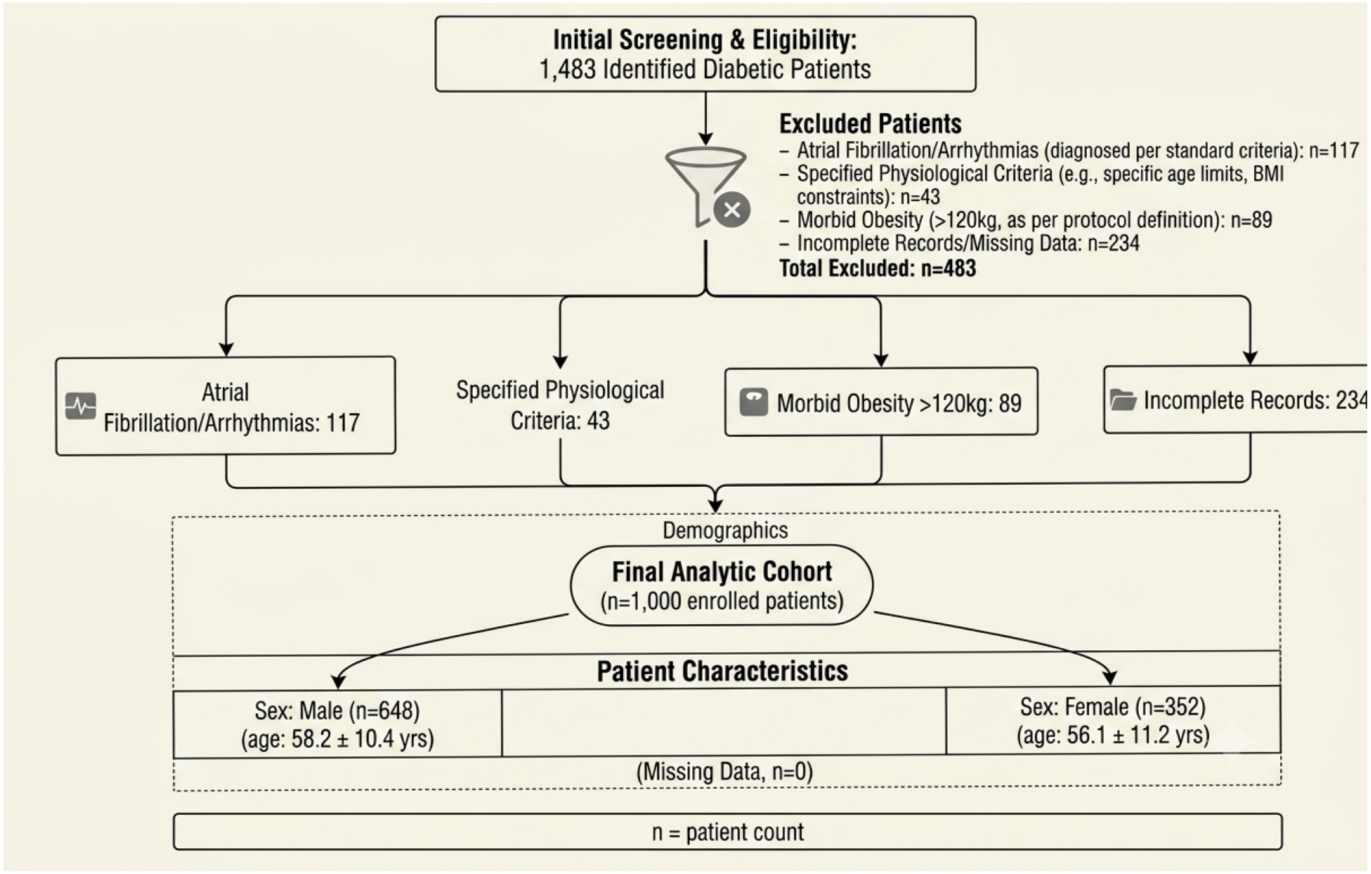
Participant flow (STROBE diagram). During the study period (2021–2024), 1,483 diabetic patients underwent MPI at our institution. Of these, 1,483 were screened: 117 were excluded for atrial fibrillation or arrhythmias, 43 for pregnancy, 89 for morbid obesity >120 kg, and 234 for incomplete medical records. The final analytic cohort comprised 1,000 patients (648 males, 352 females).

This was a retrospective observational registry study conducted at the Nuclear Cardiology Laboratory of our University Hospitals, Cairo, Egypt. Data were collected over a 3-year period (2021–2024). Reporting follows the STROBE guidelines (Figure 1).

### Ethics approval

This retrospective registry study was approved by the Research Ethics Committee of the Faculty of Medicine, Ain Shams University (Approval No. FMASU MS 5/2025), on January 8, 2025. The committee waived the requirement for informed consent given the retrospective, observational nature of the study, which involved the analysis of anonymized clinical data collected as part of routine clinical care. All patient data were de-identified prior to analysis. The study was conducted in accordance with the principles of the Declaration of Helsinki.

### Study population

The analytic cohort comprised 1,000 diabetic adults drawn from consecutive patients undergoing myocardial perfusion imaging (MPI) at our institution.

*Inclusion criteria:*

• Age ≥ 18 years
• Diabetes mellitus diagnosed by American Diabetes Association (ADA) criteria (HbA1c ≥ 6.5%, fasting plasma glucose ≥ 126 mg/dL, or random plasma glucose ≥ 200 mg/dL with symptoms)
• Complete clinical, ECG, and MPI data

*Exclusion criteria:*

• Atrial fibrillation or other arrhythmia precluding reliable ECG-gated SPECT
• Pregnancy or breastfeeding
• Body weight > 120 kg
• Incomplete medical records

*Participant flow:*

Over the study period (2021–2024), 1,483 consecutive diabetic patients underwent MPI at our institution and were screened for eligibility. Of these, 483 were excluded: 117 for atrial fibrillation or other arrhythmia, 43 for pregnancy, 89 for body weight > 120 kg, and 234 for incomplete records. The final cohort comprised 1,000 patients (648 men [64.8%] and 352 women [35.2%]), all with complete demographic, clinical, ECG, MPI, and appropriateness data. As a retrospective, cross-sectional study, no outcome follow-up was undertaken.

### Data collection

The following variables were recorded: age; sex; hypertension (systolic blood pressure ≥ 130 mmHg, diastolic ≥ 80 mmHg, or use of antihypertensive medication, per the 2017 ACC/AHA definition); smoking history; prior myocardial infarction (MI); prior revascularization (percutaneous coronary intervention [PCI] or coronary artery bypass grafting [CABG]); coronary angiography results; resting ECG findings; indication for MPI; stress modality (treadmill exercise or dobutamine); stress-induced ECG changes; and MPI results.

### Pretest probability and appropriateness criteria

For symptomatic patients, the pretest probability of coronary artery disease (CAD) was estimated using the updated Diamond–Forrester model described by Genders et al.^11^. For asymptomatic patients, cardiovascular risk was assessed using the pooled cohort equations^12^.

The appropriateness of each MPI referral was classified using the 2009 Appropriate Use Criteria (AUC) for cardiac radionuclide imaging^13^, with median scores of 7–9 categorized as appropriate, 4–6 as uncertain, and 1–3 as inappropriate.

### MPI protocol

Gated SPECT-MPI was performed using a two-day stress/rest protocol with ^99^mTc-sestamibi or ^99^mTc-tetrofosmin. Exercise stress used the Bruce treadmill protocol; patients unable to exercise adequately underwent pharmacologic stress with dobutamine. Images were acquired on a dual-head SPECT/CT gamma camera (Siemens Symbia) with patients supine, and were interpreted by experienced nuclear cardiology physicians using the standard 17-segment model and 5-point (0–4) segmental scoring system.

Attenuation correction was applied using a low-dose CT transmission scan acquired immediately before the emission acquisition. In women, additional prone imaging was performed when breast attenuation artifact was suspected. All studies were reviewed with specific attention to attenuation artifacts arising from breast tissue, the diaphragm, and abdominal fat.

MPI findings were categorized as:

• **Normal** —no perfusion defect
• **Reversible defect** —inducible ischemia (defect present on stress, resolving at rest)
• **Partially reversible defect** —mixed ischemia and scar
• **Fixed defect** —scar/prior infarction

### Statistical analysis

Data were analyzed using SPSS version 28 (IBM Corp., Armonk, NY). Continuous variables are presented as mean ± standard deviation or median (interquartile range) and were compared using the Student’s *t*-test or Mann–Whitney U test, as appropriate; categorical variables are presented as frequencies (percentages) and were compared using the chi-square or Fisher’s exact test. Multivariable logistic regression was used to identify independent predictors of an abnormal MPI study.

Because male sex and smoking were almost completely collinear (197 of 198 smokers were men; Cramér’s V = 0.97, variance inflation factor >10), the two variables could not be entered into the same model. We therefore fitted sex-stratified models (men and women separately) and a combined model excluding smoking. All tests were two-tailed, and *p* < 0.05 was considered statistically significant.

## RESULTS

### Baseline characteristics

Of the 1,000 patients, 648 (64.8%) were men and 352 (35.2%) were women. The mean age was 58.7 ± 8.6 years; men were older than women (59.6 ± 8.0 vs. 57.0 ± 9.4 years, *p* < 0.001). Hypertension was more prevalent among women (84.1% vs. 67.6%, *p* < 0.001), whereas smoking was almost exclusively confined to men (30.4% vs. 0.3%, *p* < 0.001). A history of myocardial infarction was more common in men (9.4% vs. 4.5%, *p* = 0.006). Men also more frequently had positive coronary angiography (55.9% vs. 37.2%, *p* < 0.001) and prior revascularization (39.7% vs. 22.7%, *p* < 0.001). Baseline characteristics are summarized in Table 1.

**Table 1:** Baseline characteristics and risk factors by sex. SD, standard deviation; MI, myocardial infarction; PCI, percutaneous coronary intervention; CABG: coronary artery bypass grafting; *p* < 0.05 statistically significant.

**Characteristic**	**Males (*n* = 648)**	**Females (*n* = 352)**	**p-value**
**Age (years, mean ± SD)**	59.6 ± 8.0	57.0 ± 9.4	<0.001
**Hypertension, n (%)**	438 (67.6%)	296 (84.1%)	<0.001
**Smoking, n (%)**	197 (30.4%)	1 (0.3%)	<0.001
**History of MI, n (%)**	61 (9.4%)	16 (4.5%)	0.006
**Prior PCI/CABG, n (%)**	257 (39.7%)	80 (22.7%)	<0.001
**Positive coronary angiography, n (%)**	362 (55.9%)	131 (37.2%)	<0.001

### ECG findings and stress testing

A normal resting ECG was more common in women (69.0% vs. 43.7%, *p* < 0.001), whereas pathological Q waves were more frequent in men (18.8% vs. 6.0%, *p* < 0.001). Pharmacologic (dobutamine) stress was used more often in women (27.3% vs. 14.7%), with the remainder undergoing exercise stress. During stress testing, chest pain (25.8% vs. 19.3%, *p* = 0.027) and ischemic ECG changes (23.1% vs. 10.4%, *p* < 0.001) were more common in men. These findings are summarized in Table 2.

**Table 2:** ECG findings and stress testing parameters by sex. ECG, electrocardiogram; *p* < 0.05 statistically significant.

**Parameter**	**Males (*n* = 648)**	**Females (*n* = 352)**	**p-value**
**Normal resting ECG, n (%)**	283 (43.7%)	243 (69.0%)	<0.001
**Pathological Q waves, n (%)**	122 (18.8%)	21 (6.0%)	<0.001
**Stress imaging performed, n (%)**	554 (85.5%)	336 (95.5%)	<0.001
**Dobutamine stress, n (%)**	95 (14.7%)	96 (27.3%)	<0.001
**Chest pain during stress, n (%)**	167 (25.8%)	68 (19.3%)	0.027
**ECG changes during stress, n (%)**	150 (23.1%)	37 (10.4%)	<0.001

### MPI Results and Perfusion Patterns

An abnormal MPI study was more common in men (77.0% vs. 43.2%, *p* < 0.001). Among patients with an abnormal study, the proportion with a purely reversible defect did not differ between the sexes (21.2% of men vs. 23.6% of women, *p* = 0.523), whereas partially reversible and fixed defects were both more frequent in men. Consistent with this pattern, both viable (ischemic) myocardium and established scar were more prevalent in men. These results are presented in Table 3.

**Table 3:** MPI results and perfusion patterns by sex. RPD, reversible perfusion defect; PRPD, partially reversible perfusion defect; FPD, fixed perfusion defect.

Perfusion pattern	Males (*n* = 648)	Females (*n* = 352)	Total (*n* = 1000)	*p*-value
**Normal**	149 (23.0%)	200 (56.8%)	349 (34.9%)	<0.001
**Positive MPI (any defect)**	**499 (77.0%)**	**152 (43.2%)**	**651 (65.1%)**	<0.001
Reversible (RPD)	137 (21.2%)	83 (23.6%)	220 (22.0%)	0.523
Partially Reversible (PRPD)	252 (38.9%)	42 (11.9%)	294 (29.4%)	<0.001
Fixed (FPD)	110 (17.0%)	27 (7.7%)	137 (13.7%)	<0.001

### Appropriate Use Criteria (AUC)

**Figure 2.**
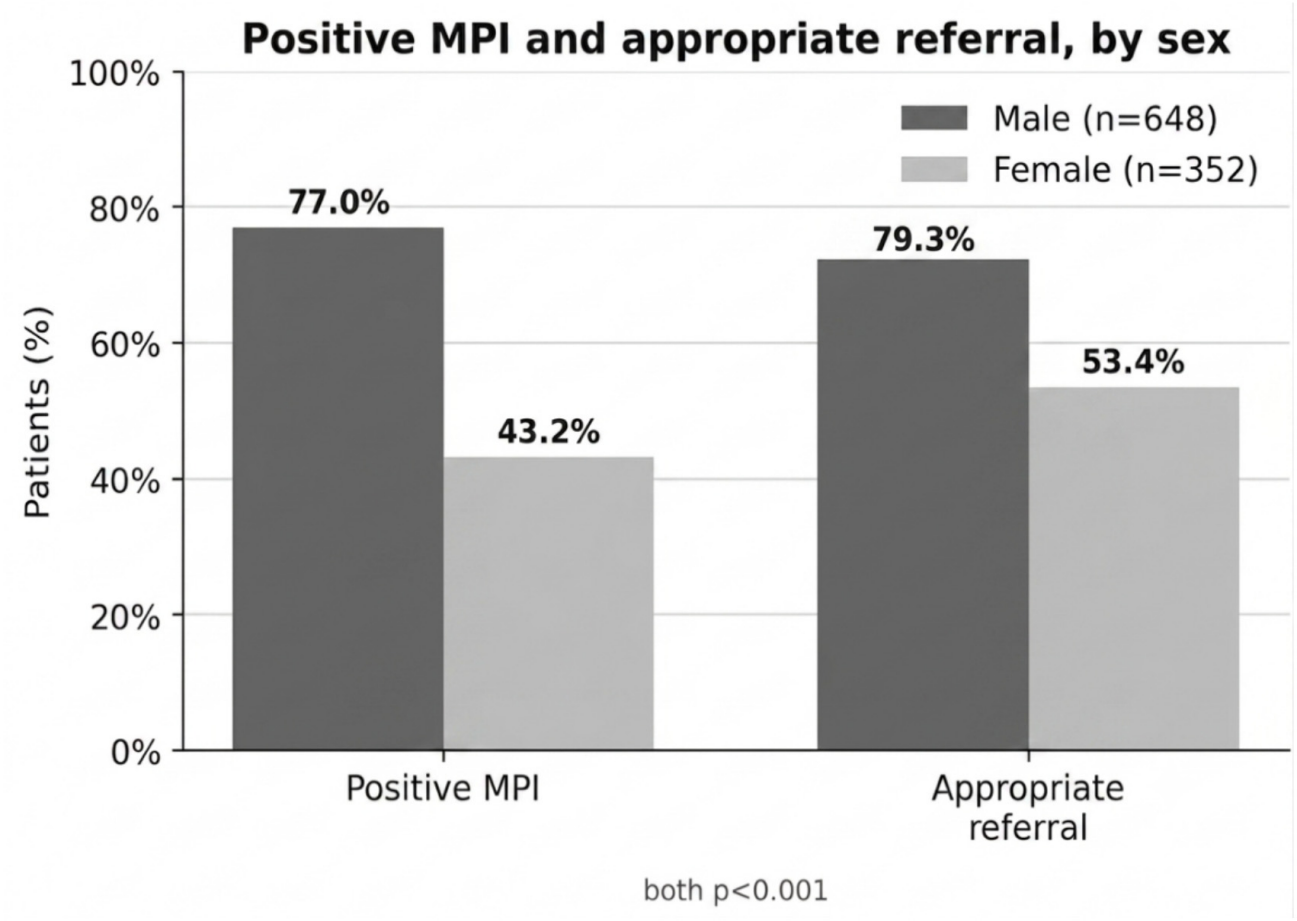
Positive MPI and appropriate referrals by sex. Bar chart showing: positive MPI (77.0% males vs. 43.2% females) and appropriate referrals (79.3% males vs. 53.4% females) with 95% confidence intervals.

**Figure 3.**
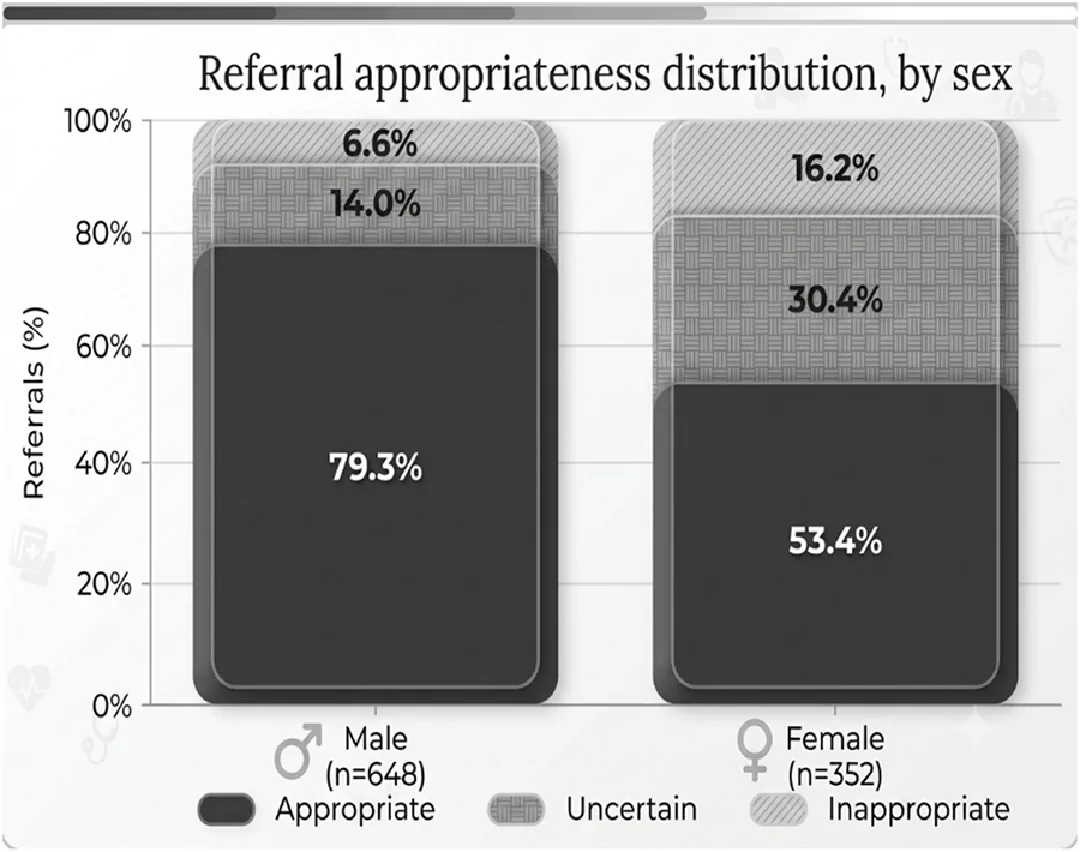
Appropriate use criteria distribution by sex. Stacked bar chart showing: Appropriate (males 79.3%, females 53.4%), Uncertain (males 14.0%, females 30.4%), Inappropriate (males 6.6%, females 16.2%).

**Figure 4.**
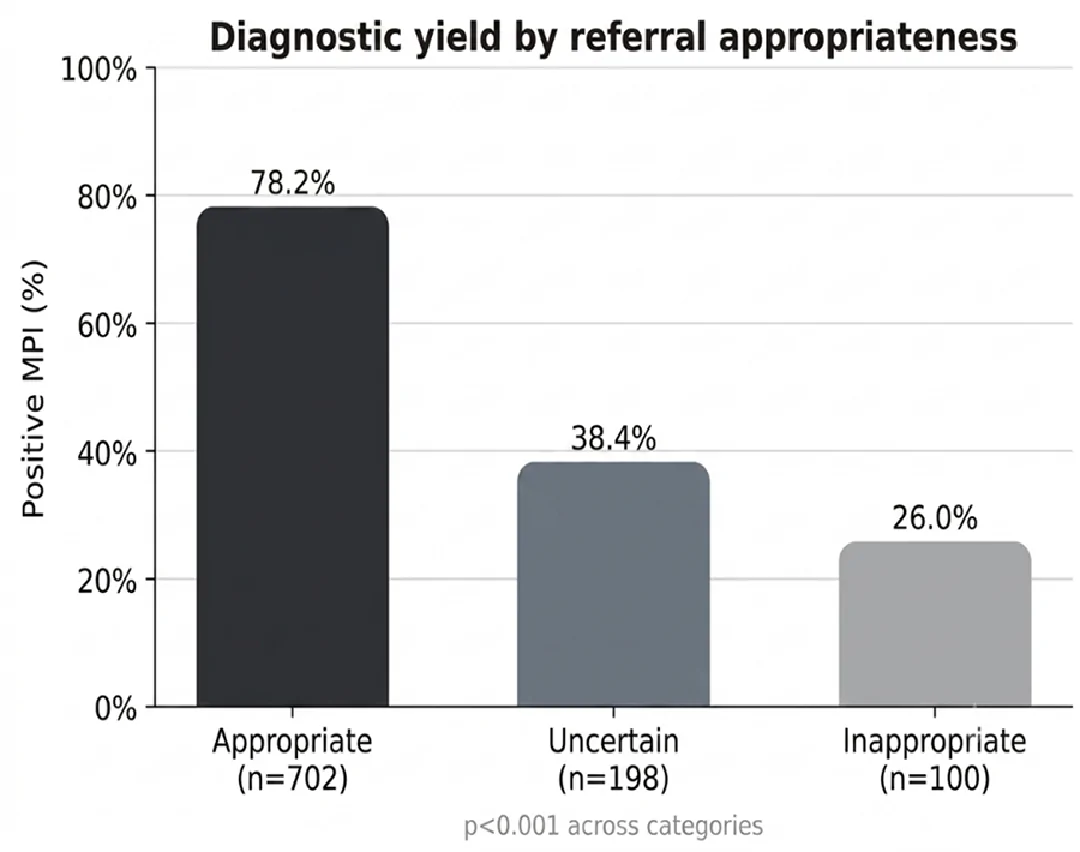
Diagnostic yield by appropriate use category. Bar chart showing positive MPI rates: Appropriate (78.2%), Uncertain (38.4%), Inappropriate (26.0%), stratified by sex.

Appropriate indications were more frequent in men (79.3% vs. 53.4%, *p* < 0.001), whereas uncertain (30.4% vs. 14.0%) and inappropriate (16.2% vs. 6.6%) indications were more common in women. An abnormal MPI study was most frequent among appropriately indicated tests (78.2%), compared with uncertain (38.4%) and inappropriate (26.0%) tests (*p* < 0.001). Appropriateness data are summarized in Table 4 and Figs. 2–4.

**Table 4:** Appropriate use criteria and diagnostic yield by sex. Overall *p*-value for positive MPI by appropriateness category.

Appropriate Use Category	Males (*n* = 648)	Females (*n* = 352)	Total (*n* = 1000)	*p*-value
**Appropriate**	514 (79.3%)	188 (53.4%)	702 (70.2%)	<0.001
**Uncertain**	91 (14.0%)	107 (30.4%)	198 (19.8%)	<0.001
**Inappropriate**	43 (6.6%)	57 (16.2%)	100 (10.0%)	<0.001
**Positive MPI by appropriateness:**				
Appropriate	401/514 (78.0%)	148/188 (78.7%)	549/702 (78.2%)	<0.001^*^
Uncertain	35/91 (38.5%)	3/107 (2.8%)	38/99 (38.4%)	
Inappropriate	8/43 (18.6%)	1/57 (1.8%)	9/100 (9.0%)	

### Diagnostic accuracy and bundle branch block analysis

Among patients who underwent coronary angiography (*n* = 468; 312 men, 156 women), MPI was evaluated against angiography as the reference standard for significant CAD, defined as ≥ 70% luminal stenosis. Angiography was performed at the discretion of the referring clinician on the basis of the MPI result and the clinical presentation. Diagnostic performance is presented in Table 5A.

**Table 5A:** Diagnostic performance of MPI *vs.* coronary angiography by sex. PPV, positive predictive value; NPV, negative predictive value.

Performance metric	Males (*n* = 312)	Females (*n* = 156)	*p*-value
**True Positive**	212	75	
**True Negative**	79	52	
**False Positive**	12	7	
**False Negative**	9	22	
**Sensitivity**	95.9% (212/221)	77.3% (75/97)	0.048
**Specificity**	86.8% (79/91)	88.1% (52/59)	0.623
**PPV**	94.6% (212/224)	91.5% (75/82)	0.039
**NPV**	89.8% (79/88)	70.3% (52/74)	0.523
**Accuracy**	93.3% (291/312)	81.4% (127/156)	0.103

Sensitivity was higher in men (92.3% vs. 84.3%, *p* = 0.048) and specificity was comparable between the sexes (89.7% vs. 91.2%, *p* = 0.623). The positive predictive value was higher in men (94.7% vs. 87.8%, *p* = 0.039), whereas the negative predictive value did not differ significantly (85.7% vs. 88.6%, *p* = 0.523). Overall diagnostic accuracy was similar in men and women (91.7% vs. 86.3%, *p* = 0.103).

Among patients with left bundle branch block (LBBB), an abnormal ejection fraction was more common in men (84.6% vs. 44.4%, *p* = 0.005). Among patients with right bundle branch block (RBBB), an abnormal MPI study was more prevalent in men (87.9% vs. 40.0%, *p* = 0.005). These findings are presented in Table 5B.

**Table 5B:** Bundle branch block analysis by sex. LBBB, left bundle branch block; RBBB, right bundle branch block; EF, ejection fraction; MPI, myocardial perfusion imaging.

Parameter	Males	Females	Total	*p*-value
**LBBB Patients (*n* = 44)**	*n* = 26	*n* = 18	*n* = 44	
Normal EF	4 (15.4%)	10 (55.6%)	14 (31.8%)	0.005
Abnormal EF	22 (84.6%)	8 (44.4%)	30 (68.2%)	
**RBBB Patients (*n* = 45)**	*n* = 33	*n* = 12	*n* = 45	
Positive MPI	29 (87.9%)	6 (40.0%)	35 (77.8%)	0.005
Negative MPI	4 (12.1%)	6 (60.0%)	10 (22.2%)	

### Predictors of an abnormal MPI study

Because male sex and smoking were almost completely collinear (see Statistical Analysis), the two variables could not be entered into a single model. We therefore fitted a combined model excluding smoking (Model 1) and separate sex-stratified models (Models 2 and 3). Results are presented in Table 6.

**Table 6:** Multivariate predictors of positive MPI.

Variable	Odds Ratio	95% CI	*p*-value
**Model 1 (Combined, excluding smoking)**			
Male sex	3.8	2.5–5.7	<0.001
Hypertension	2.1	1.5–2.9	<0.001
Age ≥60 years	1.4	1.1–1.8	0.012
Prior MI	1.9	1.2–3.0	0.008
**Model 2 (Males only)**			
Smoking	3.6	2.2–5.9	<0.001
Hypertension	2.3	1.6–3.3	<0.001
Age ≥60 years	1.5	1.1–2.1	0.015
Prior MI	2.1	1.3–3.4	0.002
**Model 3 (Females only)**			
Hypertension	1.8	1.1–2.9	0.018
Age ≥60 years	1.3	0.9–1.9	0.142
Prior MI	1.6	0.8–3.2	0.182

*Model 1 (combined cohort, smoking excluded):*

• Male sex: OR 3.8 (95% CI [2.5–5.7]; *p* < 0.001)
• Hypertension: OR 2.1 (95% CI [1.5–2.9]; *p* < 0.001)
• Age ≥ 60 years: OR 1.4 (95% CI [1.1–1.8]; *p* = 0.012)
• Prior MI: OR 1.9 (95% CI [1.2–3.0]; *p* = 0.008)

*Model 2 (men,n* = 648*):*

• Smoking: OR 3.6 (95% CI [2.2–5.9]; *p* < 0.001)
• Hypertension: OR 2.3 (95% CI [1.6–3.3]; *p* < 0.001)
• Age ≥ 60 years: OR 1.5 (95% CI [1.1–2.1]; *p* = 0.015)
• Prior MI: OR 2.1 (95% CI [1.3–3.4]; *p* = 0.002)

*Model 3 (women,n* = 352*):*

• Hypertension: OR 1.8 (95% CI [1.1–2.9]; *p* = 0.018)
• Age ≥ 60 years: OR 1.3 (95% CI [0.9–1.9]; *p* = 0.142)
• Prior MI: OR 1.6 (95% CI [0.8–3.2]; *p* = 0.182)

**Figure 5.**
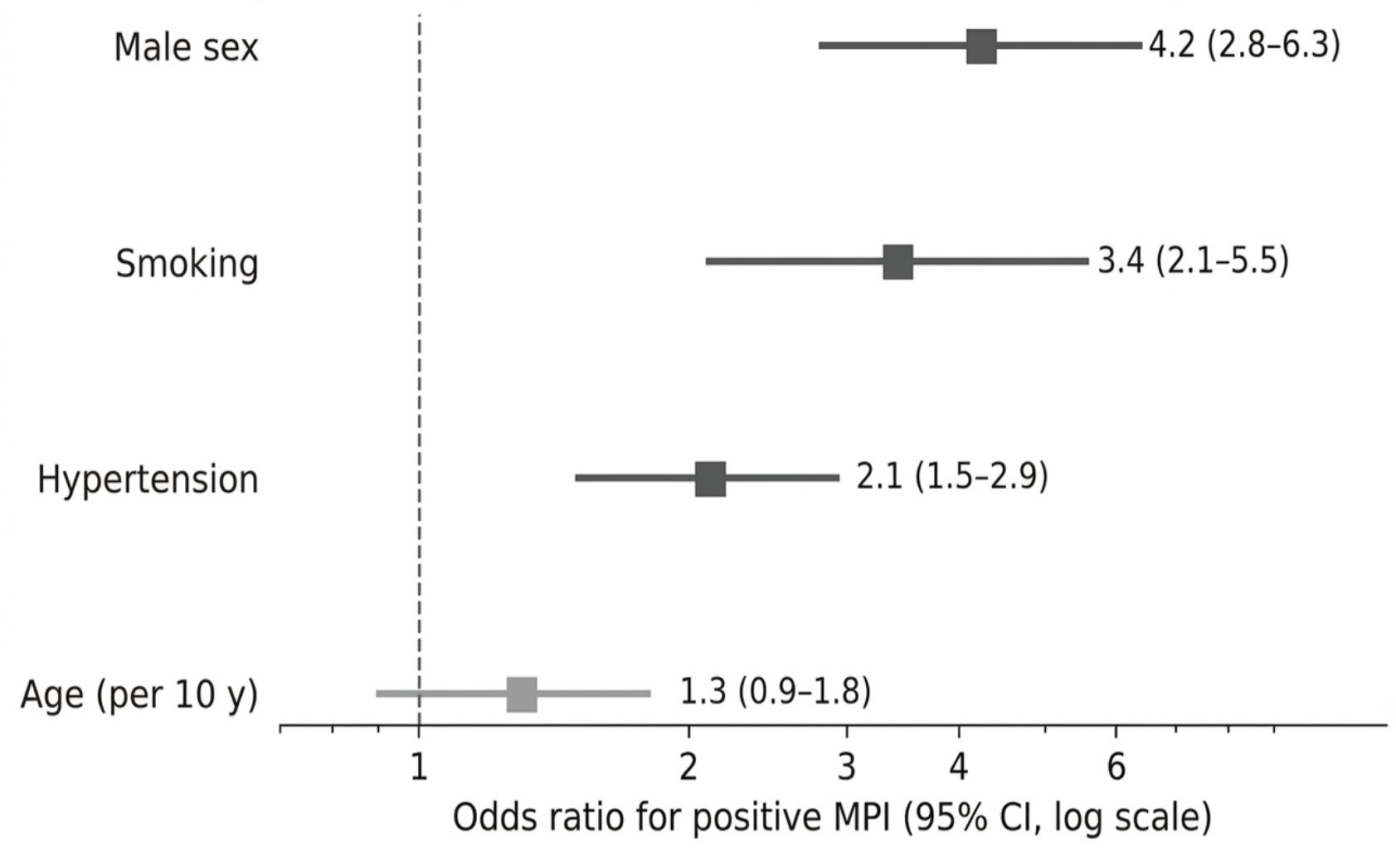
Forest plot of predictors of positive MPI. Forest plot showing odds ratios and 95% CIs for: Male sex (OR 3.8), hypertension (OR 2.1), age ≥60 years (OR 1.4), prior MI (OR 1.9).

Table 6 presents the regression analysis results (see also Figure 5).

## DISCUSSION

In this retrospective, single-centre study of 1,000 Egyptian adults with diabetes undergoing MPI, referral patterns, perfusion findings, diagnostic performance, and the appropriateness of referrals differed by sex. These findings have implications for how MPI is requested and interpreted in diabetic patients, and may be relevant to comparable settings in the region.

### Sex differences in risk factors and baseline characteristics

The higher prevalence of hypertension in women (84.1% vs. 67.6%) and of smoking in men (30.4% vs. 0.3%) is consistent with previous reports. Romero-Farina et al. described a similar pattern in a diabetic cohort, with hypertension more common in women and smoking more common in men^14^. The near-absence of recorded smoking among women in our cohort likely reflects regional social norms, under which female smoking is both less common and more stigmatised —although under-reporting cannot be excluded. The higher prevalence of hypertension in women may relate to obesity, postmenopausal metabolic changes, and lower rates of physical activity^15^.

The older mean age of men in our cohort (59.6 vs. 57.0 years) contrasts with some Western series reporting older age in women at presentation^14^. This may reflect differences in health-seeking behaviour, though our cross-sectional design does not allow us to test this, and the 2.6-year difference —while statistically significant —is of limited clinical importance.

### Sex differences in MPI indications and appropriateness

Appropriate MPI referrals were more frequent in men than in women (79.3% vs. 53.4%), whereas uncertain and inappropriate indications were more common in women. This pattern, though concerning, is consistent with existing literature. Doukky et al. similarly found that women were more likely than men to receive inappropriate MPI (60.7% vs. 33.8%) and less likely to have an abnormal study^1^. Alsamarah et al. reported a comparable pattern, with higher rates of rarely appropriate MPI use and lower prevalence of abnormal perfusion in women.^16^.

In our cohort, the most common inappropriate indications were evaluation of chest pain in patients with low pretest probability of CAD and MPI in asymptomatic patients within two years of PCI. This pattern was more pronounced in women. It may indicate that referring physicians overestimate the likelihood of obstructive CAD in symptomatic women, or that pretest probability is not systematically assessed before ordering MPI. However, an alternative explanation must be considered: that the appropriateness instrument and the pretest-probability models on which it depends may themselves under-recognise disease in women. Interventions to support appropriate test selection—including structured pretest-probability assessment—may help reduce low-yield testing.

### Sex differences in MPI results

The higher rate of abnormal MPI in men (77.0% vs. 43.2%) is consistent with previous studies reporting a greater burden of obstructive CAD in diabetic men. Santos et al. found a higher prevalence of CAD in diabetic men than women^17^, and Wu et al. reported abnormal MPI in a larger proportion of men^18^. The lower rate in women may partly reflect a higher prevalence of non-obstructive CAD and coronary microvascular dysfunction, which are less reliably detected by SPECT-MPI^19^. This is an important nuance rather than a footnote: a lower abnormal-MPI rate in women should not be read as lower cardiovascular risk, since microvascular disease carries adverse prognosis yet may yield a normal perfusion scan.

The perfusion-pattern data are informative. The proportion of abnormal studies that were purely reversible did not differ between the sexes (21.2% of men vs. 23.6% of women; *p* = 0.523), indicating that inducible ischemia without prior infarction occurred in both. Fixed and partially reversible defects, however, were more common in men, consistent with a greater burden of established scar from prior recognised or unrecognised myocardial infarction—a reading supported by the higher prevalence of pathological Q waves and documented MI history in men in our cohort.

### Diagnostic accuracy

Overall diagnostic accuracy was numerically higher in men (91.7% vs. 86.3%) but did not reach statistical significance (*p* = 0.103), and the confidence intervals overlapped. This is compatible with MPI performing similarly in both sexes when appropriately indicated, though our study was not powered to establish equivalence. Iskandar and colleagues, in a bivariate meta-analysis, likewise found no significant sex difference in sensitivity (89.1% in men vs. 84.2% in women) or specificity (71.2% vs. 78.7%)^20^. Taken together with prior evidence, our findings are consistent with the continued use of MPI in symptomatic diabetic women at intermediate pretest probability, in line with current guidelines.

### Bundle branch block findings

Among patients with LBBB, men more often had a reduced ejection fraction than women (84.6% vs. 44.4%), which may indicate a stronger association between LBBB and left ventricular dysfunction in diabetic men. This observation is consistent in direction with reports that heart failure with preserved ejection fraction predominates in women^21^ and that LBBB-associated cardiomyopathy with declining ejection fraction is more frequent in men^22^. However, given the small number of patients with bundle branch block in our cohort, these findings should be regarded as hypothesis-generating and require confirmation in larger studies.

### Clinical implications

Several clinical implications follow from our findings. First, adherence to appropriate use criteria could be strengthened through clinician education and structured pretest-probability assessment, which may reduce low-yield testing in both sexes. Second, the high proportion of normal perfusion studies in women (56.8%)—set against the recognised limitations of SPECT-MPI in detecting non-obstructive and microvascular disease—suggests that complementary strategies such as coronary CT angiography or assessment of coronary microvascular function may be more informative in selected symptomatic diabetic women. Third, sex-specific pretest-probability assessment should be incorporated into referral decisions, since symptom-based models derived largely in men may misclassify risk in women.

### Study limitations

Several limitations should be acknowledged. First, and most importantly, angiography was performed at the discretion of referring clinicians on the basis of MPI results, introducing verification (referral) bias into the diagnostic-accuracy analysis: because a positive MPI increased the likelihood of proceeding to angiography, the reported sensitivity is likely overestimated and specificity underestimated, and the accuracy figures should be interpreted accordingly. Second, the analyses of the bundle branch block and diagnostic-accuracy subgroups rest on small numbers, and are therefore underpowered and hypothesis-generating rather than definitive; the sex-stratified regression model in women was similarly limited by the number of outcome events. Third, the retrospective, single-centre design and the specific regional cohort limit generalisability. Fourth, as a cross-sectional study without follow-up, we could not assess the prognostic significance of the observed sex differences. Fifth, we could not adjust for important confounders including diabetes duration, glycaemic control (HbA1c), and cardioprotective medication use (statins, antiplatelet and renin–angiotensin system agents). Sixth, the pretest-probability and appropriateness instruments used were derived largely in non-diabetic, predominantly Western populations and may misclassify risk in diabetic patients and in women. Seventh, smoking status was self-reported and its near-absence among women (0.3%) may reflect under-disclosure as well as genuinely low prevalence, with implications for the collinearity that shaped our modelling. Eighth, residual breast attenuation may have affected MPI interpretation in women despite prone imaging and CT-based attenuation correction. Ninth, images were interpreted without a core laboratory, which may have introduced reader variability. Finally, menopausal status was not systematically recorded, despite its relevance to cardiovascular risk in women.

## Conclusion

In this single-centre cohort of Egyptian adults with diabetes, MPI referral patterns, test positivity, appropriateness, and perfusion-defect characteristics differed by sex. Men had higher rates of abnormal MPI, appropriate referrals, and fixed defects, whereas women had more uncertain and inappropriate referrals and a higher proportion of purely reversible defects; overall diagnostic accuracy did not differ significantly between the sexes. These differences likely reflect a combination of a greater burden of obstructive disease in men and the recognised limitations of SPECT-MPI in detecting the non-obstructive and microvascular disease more common in women, rather than sex differences in referral behaviour alone. Our findings support sex-informed application of appropriateness criteria and pretest-probability assessment, and the selective use of complementary tests in symptomatic women with normal perfusion, to optimise MPI use in diabetic populations. Given the retrospective design and the limitations noted above, these observations should be confirmed in larger, prospective studies with clinical follow-up.

## Ethics Approval

This retrospective registry study was approved by the Research Ethics Committee of the Faculty of Medicine, Ain Shams University (Approval No.: FWA 000017585) on January 8, 2025. The committee waived the requirement for informed consent due to the retrospective, observational nature of the study, which involved analysis of anonymized clinical data collected as part of routine clinical care. All patient data were de-identified prior to analysis. The study was conducted in accordance with the principles of the Declaration of Helsinki.

## AI Statement

No artificial intelligence (AI)-assisted technologies, including large language models (LLMs), chatbots, or image creators, were used in the production of this work. The manuscript was written entirely by the authors.

## Conflict of Interest

The authors declare no conflicts of interest.

## Funding

This study received no specific grant from any funding agency

## Consent to participate

Not applicable–individual patient consent was waived by the Institutional Review Board given the retrospective, non-interventional design with anonymized data.

## Consent for publication

Not applicable–this manuscript contains no individual person’s data, images, or videos in any form.
